# Safety and feasibility of radiofrequency ablation for treatment of Bosniak IV renal cysts

**DOI:** 10.1590/S1677-5538.IBJU.2015.0444

**Published:** 2016

**Authors:** Marcos Roberto de Menezes, Publio Cesar Cavalcante Viana, Tássia Regina Yamanari, Leonardo Oliveira Reis, William Nahas

**Affiliations:** 1 Serviço de Intervenção Guiada de Radiologia e Imagem, Instituto do Câncer do Estado de São Paulo, Universidade de São Paulo, SP, Brasil;; 2 Serviço de Intervenção Guiada de Radiologia e Imagem, Hospital Sírio Libanês, São Paulo, SP, Brasil;; 3 Departamento de Urologia, Pontifícia Universidade Católica de Campinas, PUC – Campinas, Campinas, SP, Brasil;; 4 Departamento de Urologia, Hospital das Clínicas da Faculdade de Medicina Universidade de São Paulo, SP, Brasil

**Keywords:** Safety, Feasibility Studies, Catheter Ablation, Renal cysts and diabetes syndrome [Supplementary Concept]

## Abstract

**Purpose:**

To describe our initial experience with radiofrequency ablation (RFA) of Bosniak IV renal cysts.

**Materials and Methods:**

From 2010 to 2014, 154 renal tumor cases were treated with percutaneous thermal ablation, of which 10 cases (6.4%) from nine patients were complex renal cysts and were treated with radiofrequency ablation.

**Results:**

All complex cysts were classified as Bosniak IV (four women and five men; mean age: 63.6 yrs, range: 33–83 years). One patient had a single kidney. Lesion size ranged from 1.5 to 4.1cm (mean: 2.5cm) and biopsy was performed on four cysts immediately before the procedure, all of which were malignant (two clear cell and two papillary carcinoma). Mean volume reduction of complex cysts was 25% (range: 10–40%). No patients required retreatment with RFA and no immediate or late complications were observed. The follow-up of Bosniak IV cysts had a median of 27 months (interquartile range [IQR], 23 to 38) and no recurrence or significant loss of renal function were observed.

**Conclusions:**

Mid-term follow-up of the cases in our database suggests that image-guided percutaneous RFA can treat Bosniak IV cysts with very low complication rates and satisfactorily maintain renal function.

## INTRODUCTION

Renal cell carcinoma (RCC) is the third most common malignancy of the genitourinary tract and accounts for approximately 3% of all cancers in adults. The incidence and mortality rates of RCC have increased over the past years (1) and approximately 15% of RCC cases are cystic (2). The incidental diagnosis of renal cysts has become more common as the use of advanced cross-sectional imaging techniques increases, and because some of these cysts are highly suspicious for malignancy, they must be excised based on the Bosniak classification.

The Bosniak classification of renal cysts classifies cysts as either benign or potentially malignant (complex) and is widely used to assist clinical decisions (3). Nephron-sparing approaches such as partial nephrectomy, tumor enucleation, and percutaneous ablation (e.g., cryoablation, radiofrequency, and microwave ablation) are viable options to treat these tumors while preserving renal function and lowering the risk of cardiovascular events and overall mortality (4).

A retrospective study that compared perioperative, renal functional, and oncologic outcomes of renal cryoablation and partial nephrectomy reported that postoperative glomerular filtration rate (GFR) was 6% lower than preoperative GFR in the percutaneous cryoablation group and 13% lower in the partial nephrectomy group. Additionally, this difference persisted in the multivariate analysis, but cryoablation was associated with an increased recurrence risk (5).

Percutaneous thermal ablation is an efficient and safe procedure for the treatment of small solid renal tumors (6-8), but there are two major drawbacks when treating renal cystic tumors: the possible risk of spreading tumor cells to adjacent tissues and the uncertainty of whether the procedure is effective over the entire lesion. For instance, radiofrequency ablation (RFA), in which alternating electrical current is converted into resistive heat until temperatures rise to cytotoxic levels (50–60ºC), has been well documented for the treatment of benign liver and thyroid cystic lesions (9-11).

However, few studies have investigated the use of RFA for the treatment of suspected malignant renal cysts with only short- or medium-term follow-up after RFA (12, 13).

This study describes our experience with RFA of Bosniak IV cysts with an emphasis on its feasibility, safety, and local control.

## MATERIALS AND METHODS

After institutional review board approval, we assessed our radiological database and all Bosniak IV cyst cases treated with percutaneous radiofrequency ablation (RFA) were retrospectively analyzed.

Percutaneous RFA procedures were performed in an interventional suite with CT (computer tomography) fluoroscopy capabilities under general endotracheal anesthesia using a Cool-Tip 200-W RF generator (Covidien, Mansfield, Massachusetts, USA) with single (ACT1530/ACT2030) or cluster (ACC1525) 17-gauge needle RF electrode kits.

In four cases, biopsies were conducted immediately before RFA under CT guidance, which was essential for the radiologist to position the needle inside the solid component or the septa of the lesion. An on-site pathologist confirmed that the biopsy had been adequately performed and that a sufficient amount of cells had been collected.

Prior to needle insertion, the point of entry, safe trajectory, and end position of the needle were planned with the aid of CT and the percutaneous electrode was placed inside the tumor with needle progression guided by real-time CT fluoroscopy. RFA energy was applied for 12min. Overlapping sessions were required to encompass the entire volume of the lesion with a safety margin and a CT scan was performed immediately after RFA to assess potential immediate complications.

Patients were evaluated with magnetic resonance imaging (MRI) or CT three, six, and 12 months after the procedure, and annually thereafter, to identify residual tumor and tumor recurrence. Treatment success was based on post-ablation axial imaging: increased attenuation in non-enhanced CT, reduced ablation zone size, and no enhancement on enhanced CT (<10HU) or MRI (<15% signal increase) were indicative of a successful ablation. Recurrence was defined as postablation enhancement in the ablation zone or enhancement growth on subsequent follow-up imaging.

The Clavien-Dindo classification (14) was used to report and grade the possible immediate or late complications. The immediate complications were evaluated in the CT images performed immediately after the procedure in all patients, and the late complications were analyzed by CT scan or MRI performed in the first 6 months after the ablation.

## RESULTS

From 2010 to 2014, percutaneous thermal ablation was performed in 154 renal tumor cases. Of these, 10 renal cysts (6.4%) from nine patients were Bosniak IV (four women and five men; mean age: 63.6 yrs, range: 33–83 years) and were treated with radiofrequency ablation (RFA), and one was from a single kidney patient; other comorbidities are summarized in Table-1.

**Table 1:** Patient’s age and comorbidities submitted to radiofrequency ablation.

Patient	Age	Gender	Comorbidities	Histology
**1**	60	Female	none	unknown
**2**	83	Male	colon cancer	unknown
**3**	33	Female	none	unknown
**4**	66	Female	Stroke	Papillary carcinoma
	68	Female	Stroke	unknown
**5**	64	Female	none	Clear cell carcinoma
**6**	48	Male	Chronic liver disease	unknown
**7**	72	Male	Colon cancer	unknown
**8**	75	Male	none	Papillary carcinoma
**9**	64	Male	**Single kidney**	Clear cell carcinoma

Lesion size ranged from 1.5 to 4.1cm (mean: 2.5cm) and biopsies were performed on four cysts, all of which were malignant (two clear cell and two papillary carcinomas). The others were treated based on the risk of malignancy of Bosniak IV lesions.

The location of the lesions are summarized in Table-2. Six of them were localized in places with risk of complications due to their proximity (distance <3cm) with other organs such as liver, lung or bowel, and the hydrodissection was needed and then performed. After the procedure, no complications were found.

**Table 2:** Characteristics of the Bosniak IV cysts, with location and risk of adjacent organs injury.

Lesion/Size (cm)	Location*	Organ with risk of injury (distance <3cm)	Complications
**A (3.0cm)**	Exofitic (P/S)	Adrenal and pancreas	0
**B (1.8cm)**	Central (A/M)	Coletor system	0
**C (1.5cm)**	Peripheral not exofitic (A/S)	Liver	0
**D (2.0cm)**	Exofitic (AL/I)	Colon	0
**E (4.1cm)**	Exofitic (L/M)	Liver	0
**F (3.0cm)**	Exofitic (L/M)	Colon	0
**G (2.8cm)**	Exofitic (L/MI)	-	0
**H (1.9cm)**	Exofitic (P/S)	-	0
**I (3.3cm)**	Exofitic (P/S)	-	0
**J (1.8cm)**	Exofitic (P/I)	-	0

*P = posterior; **A** = anterior; **L** = lateral; **S** = superior; **M** = middle; **I** = inferior.

The follow-up of Bosniak IV renal cysts had a median of 27 months (interquartile range [IQR], 23 to 38).

**Figure 1:**
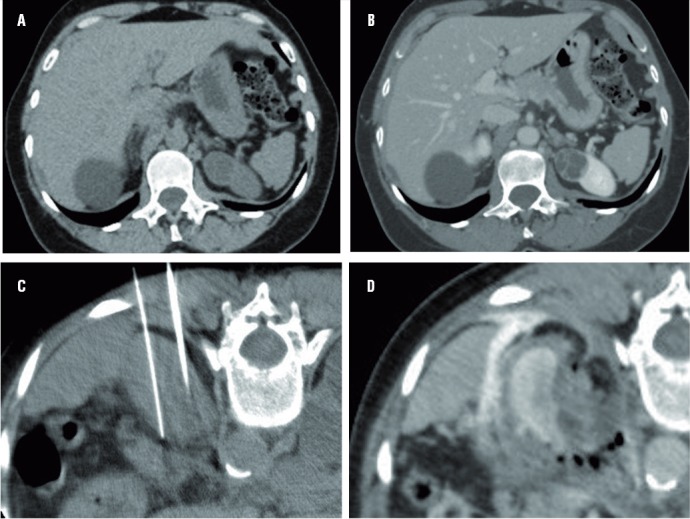
– (a-d) Tomographic images of radiofrequency ablation of Bosniak IV cystic lesion. CT without intravenous contrast (a) and post contrast (b) shows the hypoattenuation lesion in the left kidney and the exophytic cortical cyst in the upper third of the left kidney, containing thick internal septa that enhances after contrast. Non-enhanced CT (c) shows the positioning of the cluster needle in the axial plane. Enhanced-CT (d) immediately after the ablation shows the volumetric reduction of the lesion and the margins obtained after contrast injection, confirming proper treatment during the procedure.

A reduction in tumor size was observed in all Bosniak IV cyst cases immediately after the first RFA session (Figure-1). Mean volume reduction was 25% (range: 10–40%).

The procedure was successful in all cases, with CT revealing no lesion enhancement in the ablation zone immediately after the RFA, indicating that there was no residual tumor. No patients required a second RFA and no immediate or late complications were observed in this cohort.

The creatinine serum levels of each patient before and after the procedure were retrospectively assessed and the estimative of the glomerular filtration rate (GFR) was done using the Modification of Diet in Renal Disease (MDRD) formula. There was an mean variation of 10mL/min (range: 1.3–20.7) of the GFR, and no patients displayed a decrease in renal function.

**Figure 2:**
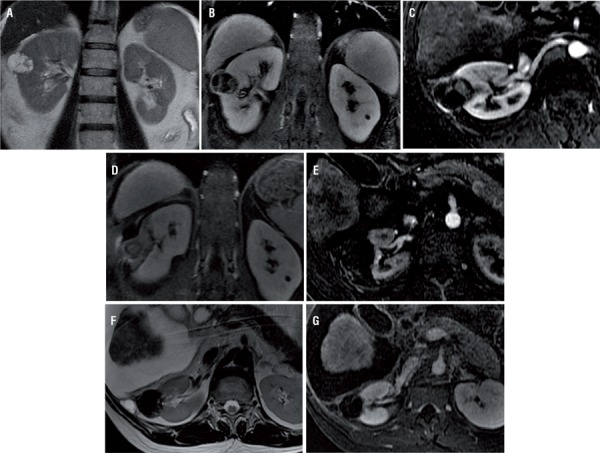
– (a-g) 48-years old man with a Bosniak IV lesion. Coronal T2-weighted (a), Coronal T1-weighted gradient echo axial MRI sequences with fat suppression (b) and coronal subtraction image (postcontrast arterial phase data - precontrast data) (c) show a cystic renal mass with thickened enhancing septa and a small solid component. Coronal T1-weighted gradient echo axial MRI sequences with fat suppression (d) performed immediately after the procedure show complete ablation of cystic lesion and no measurable enhancement within ablation zone. Axial subtraction image (postcontrast arterial phase data - precontrast data) (e) three months after the procedure show no enhancement and no recurrent of the neoplasm. Axial T2-weighted (f) and axial subtraction image (postcontrast arterial phase data - precontrast data) (g) 3 years after the procedure show ablation changes in the right kidney, without residual enhancement to suggest recurrent neoplasm.

Patients were evaluated with cross-sectional exams (contrast-enhanced CT or MRI) after the procedure, and no recurrence was found (Figure-2).

## DISCUSSION

This study describes our experience with percutaneous RFA of Bosniak IV renal cysts with an emphasis on its feasibility, safety, and local control. We showed that the procedure was effective for the treatment of Bosniak IV renal cysts. The success rate of RFA was 100%, which was confirmed by a lack of enhancement on CT and MRI scans of the treated areas within the median follow-up time of 27 months (interquartile range [IQR], 23 to 38). Our findings are in line with previous case reports and preliminary studies (12, 13, 15).

Even though Bosniak III and IV complex renal cysts are uncommon renal tumors, they are an important subgroup due to their risk of malignancy, which ranges from 16–100% and 90–100%, respectively (16). The current standard of care for both Bosniak III and IV lesions is surgical treatment. Because of their indolent growth and overall early detection, they have a better prognosis than solid tumors, and thus a more conservative approach to their treatment has been advocated. Even though ablative therapies for the treatment of small solid renal tumors are accepted options, cystic renal lesion ablation has been restricted to a few case series (12, 13).

The ablative treatment of Bosniak IV cysts is a minimally-invasive procedure that has not been yet associated with major complications to the best of our knowledge, and it leads to preservation of the renal parenchyma.

Among 10 Bosniak IV cysts of our study, four were histologically proven to be renal cell carcinomas (two clear cell subtypes and two papillary subtypes). Some theoretical questions about the treatment of Bosniak IV renal cysts still remain unanswered, especially the concern about neoplastic implants and cyst rupture caused by biopsies and therapeutic punctures. Anecdotal cases of implants related to biopsy of solid lesions have been reported, but there are no studies to date of cystic lesion implants after percutaneous biopsies or RFA treatments. We biopsied four lesions that proved to be malignant and found no neoplastic implants at follow-up. Felker et al. (15) also reported no neoplastic implants related to biopsy or treatment of 23 cystic neoplastic lesions during a 24-month follow-up period.

There were no procedure-related complications, showing that percutaneous RFA is a safe method, especially in selected cases. In our sample, almost all cases were classified as clinical stage T1a tumors, and most were predominantly exophytic, with a safe distance to the collector system and in polar location. Although there is no adequate information on the selection criteria for RFA mentioned above, previous studies have not reported clinically relevant complications either (12, 13, 15).

All biopsies and RFA procedures in our study were done under CT fluoroscopy. We believe that CT provides good visual control of the procedure. Additionally, needle trajectory was planned and controlled with Multiplan views. The radiologist was able to identify and prevent possible complications by planning the best access and performing hydrodisplacement of bowel or other structures when those were adjacent to the renal lesion. This supports the use of CT guidance over other imaging modalities such as ultrasound.

Needle positioning was CT-guided, which enabled three-dimensional visualization of the lesion. Thus, the interventional radiologist was able to plan the needle trajectory, detect possible injuries in adjacent structures, and prevent complications such as lesions in intestinal or vascular loops. Because no acute or late complications were observed in our sample, the success rate of RFA can be partly attributed to CT-planning.

To our knowledge, only three retrospective series evaluated percutaneous RFA for the treatment of renal cysts, and only one included just biopsy-proven malignant cystic disease (Table-3) (12, 13, 15).

**Table 3:** RFA of cystic renal lesions to date.

Study	Patients	Tumors	Biopsy	Size (cm)	Follow-up (months)	Efficacy	Major Complications
Park et al. 2008 (13)	9	14	0	2.5	8	100%	0
Allen et al. 2013 (12)	38	40	90% (60% cancer)	2.3	32	100%	1 (pulmonary edema)
Felker et al. 2013 (15)	16	23	100%	3.1	24	91%	0
Current, 2014	9	10	40% (100% cancer)	2.5	29	100%	0

The procedure was successful in all cases, with no lesion enhancement in the ablation zone on CT immediately after percutaneous RFA and on follow-up CT or MR exams, indicating that there was no residual tumor after the median of 27-months follow-up.

In our study, we found a shrink of tumor size in all Bosniak IV cyst cases with the mean volume reduction of approximately 25% in all lesions immediately after the RFA procedure. Because there was no recurrence during the follow-up period, the mean volume reduction could be used as an intraoperative measure of treatment success. Nevertheless, a larger sample and more studies are needed to support this finding.

Although the “heat sink effect” caused by undesired RF energy dispersion is less frequent in small cystic lesions and more evident in large, highly vascularized tissues, it represents one of the most important limitations of the RFA technique. In our sample, we treated small lesions (diameter: 1.5–4.1cm; mean: 2.5cm) and our results were not affected by this limitation. Interestingly, microwave ablation (MWA) may minimize thermal dispersion and reduce the “heat sink effect”, resulting in a positive outcome in larger cystic lesions, but only a single study to date has investigated the efficacy of MWA in only seven cystic renal lesions, which was not enough to adequately assess the efficacy of MWA (16).

The relatively small sample size, intermediate follow-up time and selection biases due to the retrospective series are limitations of our study. However, even though more comprehensive studies are needed to support the effectiveness of RFA for the treatment of Bosniak IV renal lesions, its efficacy and safety in our study were similar to those reported in the few studies available.

Another limitation of our study was the percentage of lesions submitted to biopsy prior to the RFA (40%). However, the technical difficulties of performing percutaneous biopsies of predominantly cystic lesions should be considered, because some lesions have a small solid component where the needle should be positioned for collecting material for pathological analysis. Moreover, even if no malignant cells are found in the biopsy, Bosniak IV renal cysts still can be excised based on the presumed risk of malignancy based on the Bosniak classification (17), and based on the fact that there are still few studies that demonstrate the negative predictive value of the biopsy of Bosniak IV renal cysts (18). Additionally, if the lesion is not excised after biopsy, follow-up is compromised because of parenchymal distortion and changes in cyst density and signal on CT and MRI, respectively. The other Bosniak IV cysts were not biopsied and therapeutic indication was supported by presumed malignancy based on the Bosniak classification.

In conclusion, despite the limitations of our study, the results suggest that image-guided percutaneous RFA can properly treat Bosniak IV renal cysts with very low complication rates and satisfactorily maintain renal function.
